# Ultrasound-assisted preparation of lactoferrin-EGCG conjugates and their application in forming and stabilizing algae oil emulsions

**DOI:** 10.1016/j.ultsonch.2022.106110

**Published:** 2022-08-01

**Authors:** Sairui Zhang, Xueqi Li, Xiaojia Yan, David Julian McClements, Cuicui Ma, Xuebo Liu, Fuguo Liu

**Affiliations:** a College of Food Science and Engineering, Northwest A&F University, Yangling 712100, Shaanxi, China; b Department of Food Science, University of Massachusetts Amherst, Amherst, MA 01003, USA

**Keywords:** Ultrasonic treatment, EGCG, Lactoferrin, Algae oil, Nanoemulsions, Oxidative stability

## Abstract

The aim of this study was to prepare lactoferrin-epigallocatechin-3-gallate (LF–EGCG) conjugates and to determine their ability to protect emulsified algal oil against aggregation and oxidation. LF–EGCG conjugates were formed using an ultrasound-assisted alkaline treatment. The ultrasonic treatment significantly improved the grafting efficiency of LF and EGCG and shortened the reaction time from 24 h to 40 min. Fourier transform infrared spectroscopy and circular dichroism spectroscopy analyses showed that the covalent/non-covalent complexes could be formed between LF and EGCG, with the C=O and C—N groups playing an important role. The formation of the conjugates reduced the α-helix content and increased the random coil content of the LF. Moreover, the antioxidant activity of LF was significantly enhanced after conjugation with EGCG. LF–EGCG conjugates as emulsifiers were better at inhibiting oil droplet aggregation and oxidation than LF alone. This study demonstrates that ultrasound-assisted formation of protein–polyphenol conjugates can enhance the functional properties of the proteins, thereby extending their application as functional ingredients in nutritionally fortified foods.

## Highlights

• Lactoferrin (LF) − EGCG conjugates were prepared using an ultrasound-assisted method.
• The antioxidant properties of the conjugates were better than that of pure LF.
• The conjugates were used to stabilize algae oil-in-water emulsions.
• The oxidative stability of the emulsions was enhanced using the conjugates.

## Graphical abstract

**Figure:**
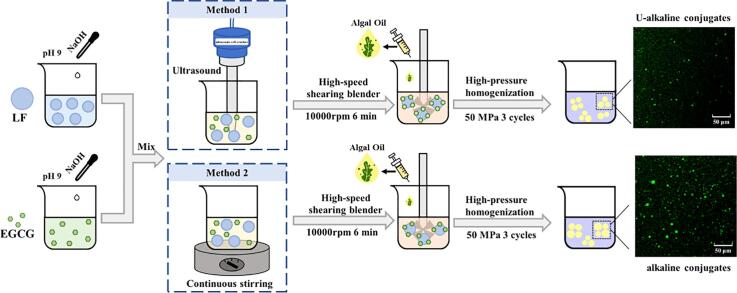

## 1 Introduction

The modern food industry increasingly focuses on foods that are specifically designed to improve human health and wellbeing. For this reason, bioactive ingredients derived from animals and plants, such as polyunsaturated fatty acids and polyphenols, are being incorporated into functional foods as potentially health promoting ingredients [1], [2]. Docosahexaenoic acid (DHA), an omega-3 polyunsaturated fatty acid, is widely used in food and supplement products due to its potential health effects, including inhibiting cardiovascular disease [3], improving eyesight [4], and promoting the development of infants and young children [5]. However, the high hydrophobicity and chemical reactivity of DHA means that it has a low water-dispersibility, is susceptible to degradation during food processing, storage, and distribution, and often has a low bioavailability after ingestion, which restricts its application as a functional food ingredient [6]. Emulsion-based delivery systems are therefore being explored to improve the dispersibility, stability, and bioavailability of these kinds of functional lipids [7]. The properties of the interfacial membrane coating the fat droplets in emulsions is known to be an important factor in determining their physicochemical stability and gastrointestinal fate [8], [9].

Many food-grade proteins are natural emulsifiers that can be used to form and stabilize emulsions because they can adsorb to the surfaces of oil droplets and form a protective membrane around them [10], [11]. In some cases, proteins can also act as interfacial antioxidants because they can inhibit lipid oxidation when they adsorb to oil–water interfaces [12]. Consequently, they can be used as dual-purpose functional ingredients: emulsifiers and antioxidants. However, the antioxidant activity of many food proteins is rather limited. Studies have shown that the antioxidant activity of proteins can be enhanced by forming covalent or non-covalent complexes with natural antioxidant polyphenols, such as tea polyphenols [13], [14]. The attachment of the polyphenols to the proteins brings them close to the oil–water interface, which is the site where lipid oxidation usually occurs in emulsions [9], [15].

Protein–polyphenol complexes are formed through non-covalent interactions such as hydrogen bonding, hydrophobic interactions, or electrostatic attraction [16], [17]. In contrast, protein–polyphenol conjugates are normally formed through covalent interactions between protein and polyphenol molecules. The alkaline conjugation method is commonly used for this purpose because it is simple, reliable, and economic to perform [16]. Studies have shown that compared to pure proteins, protein–polyphenol conjugates have better thermal stability and antioxidant activity [18], [19]. As a result, conjugation may increase the utilization of proteins as functional ingredients within foods.

One of the major drawbacks of using the traditional alkaline method for forming protein–polyphenol conjugates is the relatively long reaction time required, which is a hurdle to the large-scale commercial production of these functional ingredients. In principle, this limitation can be overcome by combining the alkaline method with an ultrasound-assisted treatment. Sonication is known to promote protein unfolding through a combination of mechanical, cavitation, and thermal effects, which can increase the ability of proteins to be covalently attached to other molecules [20], [21]. A previous study have shown that sonication can accelerate the reaction between peanut protein isolate (PPI) and polysaccharides (dextran or gum Arabic), thereby shortening the reaction time and increasing the grafting degree [22]. There have also been some examples of improved conjugation of proteins and polyphenols using sonication. For instance, Jing et al. [23] used an ultrasound-assisted alkaline/free radical treatment to prepare egg white protein-tea polyphenol (EWP-TP) conjugates. The ultrasound-assisted treatment improved the grafting efficiency of the protein and polyphenol and shortened the reaction time from 24 to 1 h. To the authors knowledge, however, there have been few previous reports on the application of protein–polyphenol conjugates fabricated by an ultrasound-assisted alkaline treatment as interfacial antioxidants in emulsions.

Therefore, in this study, lactoferrin-epigallocatechin-3-gallate (LF–EGCG) conjugates were fabricated using an ultrasound-assisted alkaline treatment. The aim of this article is to explore the effects of ultrasonic treatment and EGCG modification on the structure and antioxidant properties of LF. In addition, the ability of LF–EGCG conjugates and complexes to protect algae oil droplets from aggregation and oxidation was investigated. The results of this research may lead to the creation of a new class of dual-function ingredients (antioxidant emulsifiers) that can be used to improve the stability of foods containing polyunsaturated lipids.

## 2 Materials and methods

### 2.1 Materials

Lactoferrin (LF, purity > 96 %) was obtained from Tatua Cooperative Dairy Co., Ltd. (Tatuanui, New Zealand). Epigallocatechin-3-gallate (EGCG, purity > 98 %) was purchased from BSZH Science Company (Beijing, China). Algal oil (DHA > 45 %) was donated by Qingdao Xunon Bioengineering Co., Ltd (Qingdao, China). Folin−Ciocalteu reagent was purchased from Solarbio Company (Beijing, China). 1,1-diphenyl-2-picrylhydrazyl (DPPH, purity 95 %) was purchased from Sigma-Aldrich Company (St. Louis, MO, USA). 1,1,3,3-Tetraethoxypropane (purity > 97 %) was provided by Shanghai Aladdin Biochemical Technology Co., Ltd (Shanghai, China). 2-Thiobarbituric acid was purchased from Shanghai Macklin Biochemical Co., Ltd (Shanghai, China). All other chemicals used were of analytically grade.

### 2.2 Preparation of LF–EGCG conjugates

The LF–EGCG conjugates were fabricated using an ultrasound-assisted alkaline treatment according to a previously reported method with slight modifications [24], [25]. Briefly, an LF (2 wt%) solution and a EGCG (0.4 wt%) solution were prepared by dissolving the powdered ingredients in distilled water and then adjusting the pH to 9.0 using 0.5 M NaOH. Then, the EGCG solution was mixed with the LF solution under continuous stirring (200 rpm), and the mixture was readjusted to pH 9.0 using 0.5 M NaOH. The mixture was sonicated (300 W, 3/2 s on/off) for different time (10, 20, 30, 40, or 60 min) using an ultrasonic cell (Shanghai Huxi Industrial Co., ltd.). Since the ultrasonic cell crusher used in this study did not have a temperature control device, an external water bath was used to avoid overheating of the samples during sonication. Subsequently, the samples were dialyzed at 4 °C for 48 h, with the water being changed every 6 h. After freeze-drying, the samples were placed in a desiccator and stored. The conjugates received under different high-intensity ultrasonic treatments are referred to as U10, U20, U30, U40, or U60, with the number referring to the sonication time in minutes. The samples with the highest EGCG equivalents were referred to as U-alkaline conjugates in the subsequent experiments.

Alkaline conjugates were fabricated by ordinary alkaline treatment using the same steps discussed above but without sonication [18]. The mixed solution was maintained at 25 °C with continuous stirring (200 rpm) for 24 h with full exposure to the air. After dialysis, the samples were lyophilized and labeled as alkaline conjugates. Native LF, ultrasound-treated LF, and physical mixtures of LF and EGCG were used as controls, which were labelled as LF, U-LF, and physical complexes, respectively.

### 2.3 Measurement of total EGCG content in the conjugates

The total EGCG content of the samples was measured using the Folin-Ciocalteu method with some modifications [26]. In brief, 0.5 mL of sample (0.5 mg/mL) was mixed with 2.5 mL of Folin-Ciocalteu reagent (0.2 N). After 30 s vortex, the mixture was incubated in the dark for 7 min. Subsequently, 2 mL of Na_2_CO_3_ (7.5 %, w/v) was added, and the mixture was allowed to rest at room temperature for 2 h in the dark. A UV–visible spectrophotometer (UV-1240, Shimadzu, Japan) was used to measure the absorbance of the mixture at 760 nm against a control solution consisting of an LF solution prepared using the same procedure. The total polyphenol content of the different samples was expressed as equivalent content of EGCG using a calibration equation (*Abs* = 9.0843 *[EGCG]* + 0.0131, R^2^ = 0.9990) obtained by measuring the absorbances of solutions containing different EGCG concentrations (0.2, 0.4, 0.6, 0.8, 1.0 mg/mL). The results are expressed as mg EGCG g^−1^ sample.

### 2.4 Sodium dodecyl sulfate–polyacrylamide gel electrophoresis (SDS-PAGE)

SDS-PAGE was carried out according to the method described by Li et al. [27] with some modifications. The stacking and separating gels were prepared using 4 % and 8 % acrylamide, respectively. To begin with, the samples were dissolved in distilled water to obtain a concentration of 0.33 mg/mL and then mixed with a reducing reagent at 4:1. The thickness of the vertical slab gel was 1 mm, the loading volume of each mixed solution was 9 μL, and the protein standard solution was 2.5 μL. The voltage was 80 V for the first 30 min and then increased to 120 V until the end. Finally, the gel was stained with Coomassie Brilliant Blue R-250 for 15 min and faded using decolorizing liquid (methanol: glacial acetic acid: water = 1:1:8) until the protein bands were visible.

### 2.5 Fourier-transform infrared (FTIR) spectroscopy

The infrared spectra of the samples were acquired using a FTIR Spectrometer (Vector 70, Bruker, Germany). In brief, the freeze-dried samples were mixed with potassium bromide (KBr) at a ratio of 1:100, ground into a uniform powder in an agate mortar with a pestle, and then pressed into a pellet for scanning. Pure KBr was used as a blank control. The spectra were scanned ranging from 400 to 4000 cm^−1^ with 32 scanning time at a resolution of 4 cm^−1^.

### 2.6 Circular dichroism (CD) spectroscopy

The circular dichroism spectrometer (ChirascanV100, Applied Photophysics Limited, U.K.) was used to measure the secondary structure of the samples using the method described by Ma et al. [28] with slight modifications. A quartz cuvette with a path length of 1 mm was used to scan the sample solution (0.2 mg/mL) in the range from 190 to 260 nm under continuous nitrogen flow at room temperature. The operating parameters were set to a scan speed of 120 nm/min, a bandwidth of 1.0 nm, and a path length of 0.5 nm. Each spectrum was scanned three times and then averaged to reduce the effect of background noise. The secondary structure of the samples was analyzed using an online program (Dichroweb) as described previously [26].

### 2.7 DPPH free radical scavenging activity

The DPPH free radical scavenging activity of the samples was determined according to the method described by Li et al. [27] with some modifications. In short, 2 mL of each sample solution (0.05 mg/mL) was mixed with 2 mL of DPPH solution (0.125 mM ethanol) and then the mixture was incubated in the dark at room temperature for 30 min. After that, the absorbance at 517 nm was determined using a UV–visible spectrophotometer (UV-1240, Shimadzu, Japan), and the DPPH free radical scavenging rate was calculated using the following equation:

$$DPPH\;free\;radical\;scavenging\;rate\;\left(\%\right)=\left[A_{0}-\left(A_{2}-A_{1}\right)\right]/A_{0}\times 100$$

Here, $A_{0}$, $A_{1}$, and $A_{2}$ represent the absorbances of the mixture of distilled water and DPPH solution, ethanol and sample solution, and DPPH and sample solution, respectively.

### 2.8 Emulsion preparation

The emulsions were prepared using a method described by Ma et al. [29] with some adjustments. An aqueous phase was prepared by dispersing 8 mg/mL of emulsifier (LF, U-LF, physical complexes, U-alkaline conjugates, or alkaline conjugates) in deionized water, and then stirring overnight at room temperature. Algal oil was added to the aqueous phase while stirring at 300 rpm, then a coarse oil-in-water emulsion was formed by blending 10 % oil (v/v) and 90 % water (v/v) using a high-speed shearing blender (Ultra-Turrax IKA-T25, Staufen, Germany) at 10,000 rpm for 6 min. Subsequently, a fine emulsion was produced by passing the coarse emulsion through a high-pressure homogenizer (AH-BASIC, ATS Engineering Company, China) at 50 MPa for 3 cycles.

### 2.9 Droplet surface potential

The effective surface potential (zeta-potential) of the emulsifier-coated oil droplets was measured using particle electrophoresis (Malvern Zetasizer Nano ZS, ZEN3600, Malvern Instruments, Worcestershire, U.K.). To avoid multiple scattering effects, the sample solutions were diluted using distilled water with the same pH.

### 2.10 Confocal laser scanning microscopy (CLSM)

Changes in the structure and organization of the different components in the emulsions during storage were observed using confocal laser scanning microscopy (LEICA TCS SP8, Germany), according to a method described previously [30]. Briefly, 30 μL of emulsion samples was placed onto a glass microscope slide. After being stained with a mixture of Fast green (for protein staining, 0.1 % w/v in distilled water) and Nile red (for oil phase staining, 0.1 % w/v in DMSO) at a ratio of 1:1, a coverslip was placed on top. Two laser excitation sources (488 and 633 nm) and two acquisition channels for Nile red and Fast green were used, respectively. Images were acquired at a 40× (objective lens) magnification using a He-Ne laser.

### 2.11 Lipid oxidation stability of emulsions

The resistance of the samples to oxidation was obtained by placing 1.8 mL of emulsion into a 2 mL centrifuge tube and then storing in the dark at 25 or 37 °C for 12 days. The hydroperoxide and TBARS values were measured every 3 days, and the specific measurement methods are as follows.

#### 2.11.1 Lipid hydroperoxides

Lipid hydroperoxides were measured according to a method described by Zhu et al. [31] with some adjustments. First, 0.3 mL of emulsion was added to 1.5 mL of isooctane/2-propanol mixture (3:1, v/v) and vortexed for 10 s, which was repeated 3 times. After centrifugation at 9000 rpm for 3 min, the supernatant was collected in a 1.5 mL centrifuge tube for later use. Second, 2.8 mL of methanol/1-butanol (2:1, v/v), *x* mL of organic layer, and (0.2-*x*) mL of water was added into a 4 mL centrifuge tube. After mixing, the mixture was reacted with 30 μL of the mixed solution of 3.94 M ammonium thiocyanate and Fe^2^^+^ solution (prepared by mixing 0.132 M barium chloride and 0.144 M ferrous sulfate at a 1:1 (v/v) ratio) at a ratio of 1:1 (v/v). The mixture was vibrated and incubated in the dark for 20 min at room temperature, after which the absorbance of the sample mixture was measured at 510 nm. Different cumene hydroperoxide concentrations were prepared and measured using the same procedures to obtain a standard curve, which was used to calculate the concentration of hydroperoxide in the sample.

#### 2.11.2 TBARS

The TBARS concentration of the samples was determined using a method described by Zhu et al. [31] with some adjustments. Briefly, *x* mL of emulsion and (1.5-*x*) mL of water was added to a 10 mL centrifuge tube, then 3 mL of TBA-TCA working solution (prepared by mixing 5 g of trichloroacetic acid, 414 g of water, 8.8 mL of 12 M HCl, and 1.99 g of TBA) was added. 10 mL centrifuge tubes containing 4.5 mL of the mixtures were placed in a boiling water bath for 15 min and cooled down in a cold water bath to room temperature for 10 min. After passing through a 0.55 μm filter, the absorbance of the filtrate was measured at 532 nm. The TBARS concentration was determined using a standard curve that was obtained by measuring the absorbance of a series of solutions containing different 1,1,3,3-tetraethoxypropane (TEP) levels (2.5, 5, 7.5, 10, 12.5, and 15 μmmol/L) using the same procedure mentioned before. The standard result is *Abs* = 0.0478 *[TEP]* + 0.0103 (R^2^ = 0.9991).

### 2.12 Statistical analysis

Each sample was measured in triplicate and the results are presented as mean ± standard deviation (SD). The significance of sample differences between groups (*p* < 0.05) was analyzed using an analysis of variance (ANOVA) with the help of the SPSS statistical analysis system. Graphs were plotted using Origin software (version 8.6).

## 3 Results and discussion

### 3.1 Formation and structure of LF–EGCG conjugates

#### 3.1.1 Total EGCG content in the conjugates

Due to the multiple hydroxyl groups in their chemical structure, polyphenols have high chemical reactivity, and tend to be oxidized into quinones through non-enzymatic pathways easily, which can react with nucleophilic groups of protein side chains such as –NH_2_ and -SH groups to form conjugates [16]. Higher total phenol equivalents indicate a greater extent of EGCG grafting to the LF molecules. As shown in Table 1, the polyphenol binding equivalent of the LF–EGCG conjugates increased and then decreased as the sonication time increased. The conjugates fabricated using the ultrasound-assisted treatment for 40 min had the highest total EGCG content (125.7 mg/g), which was higher than that of the physical complexes (30.7 mg/g) and showed no significant difference from the alkaline conjugates (123.6 mg/g). This result showed that the ultrasonic treatment could significantly shorten the reaction time without affecting the degree of polyphenol grafting.

**Table 1:** Total phenolic content in LF–EGCG physical complexes and LF–EGCG conjugates.^a^

**Sample Name**	**EGCG conjugation equivalent (mg/g)**
physical complexes	30.7 ± 3.4^e^
U10	95.4 ± 1.3^d^
U20	99.2 ± 6.9^d^
U30	117.8 ± 5.0^b^
U40	125.7 ± 4.3^a^
U60	107.9 ± 6.7^c^
alkaline conjugates	123.6 ± 5.4^a^

a Different lowercase letters represent a significant difference (*p* < 0.05). U10, U20, U30, U40, or U60 were the LF–EGCG conjugates fabricated by ultrasound-assisted alkaline treatment with 10, 20, 30, 40, or 60 min.

The observed increase in polyphenol equivalents during the early stages of treatment may be due to the combined effects of the disruptive energy and heat generated by sonication. Sonication could also have promoted unfolding of the protein molecules due to mechanical and cavitation effects associated with the high-intensity ultrasonic waves [32], [33], as well as due to a rise in temperature during treatment [34]. Both of these effects can increase the number of free amino groups available and cause an increase in the polyphenol equivalents of the samples during the early stages of sonication. The decrease in polyphenol equivalents observed after prolonged sonication might be due to the fact that the energy and heat generated by extended sonication promoted protein aggregation, there reducing the number of reaction sites available at the protein surfaces [34], [35].

Indeed, previous studies have shown that excessive sonication can promote denaturation and aggregation of LF, which reduced the level of polyphenols bound to the proteins [36]. In addition, excessive sonication causes water molecules to decompose into hydroxyl and hydrogen ion radicals, which promotes decomposition of the polyphenols [37]. Based on the above results, the LF–EGCG conjugates fabricated by ultrasound-assistant alkaline treatment for 40 min were chosen for the subsequent studies.

#### 3.1.2 SDS-PAGE

**Fig. 1:**
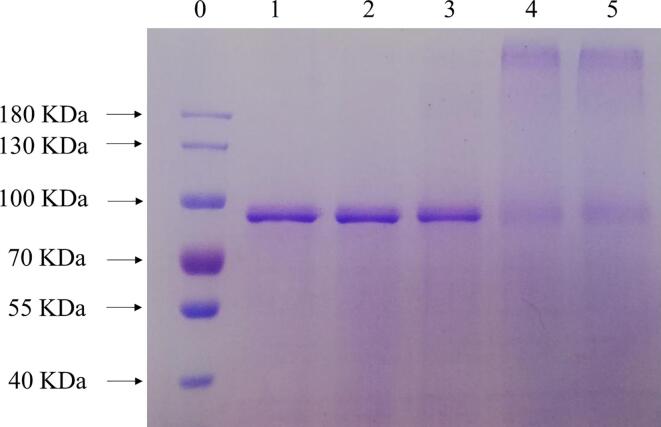
SDS-PAGE patterns of LF, U-LF, LF–EGCG physical complexes, and LF–EGCG conjugates: Lane 0 marker; Lane 1 LF; Lane 2 U-LF; Lane 3 physical complexes; Lane 4 U-alkaline conjugates; Lane 5 alkaline conjugates.

As shown in Fig. 1, the band corresponding to the LF was found to be around 80 to 90 kDa and the ultrasonic treatment had no significant effect on the molecular weight of LF, which was in accordance with previous studies [18], [21]. Compared to pure LF or U-LF, the location of the U-alkaline conjugates and alkaline conjugates migrated upwards, and the band strength of LF between 70 and 100 kDa decreased. In contrast, there was no marked change in the location and intensity of the physical complexes band. SDS is an anionic detergent that can break the hydrogen bonds within and between molecules and disrupt the secondary and tertiary structure of the protein. Therefore, these results suggest that covalent bonds formed between the EGCG and LF in the U-alkaline/alkaline conjugates. In addition, the band intensity of the conjugates at the beginning of the separation gel increased significantly, suggesting that there was a large increase in molecular weight. Since the molecular mass of EGCG is only 458.4 Da, it can not cause such a large molecular weight change connecting with a single protein. Therefore, it suggests that EGCG could act as a bridge between proteins and promote the formation of high molecular weight complexes. This phenomenon is consistent with the results of previous studies [18], [19].

#### 3.1.3 Fourier-transform infrared (FTIR) spectroscopy

**Fig. 2:**
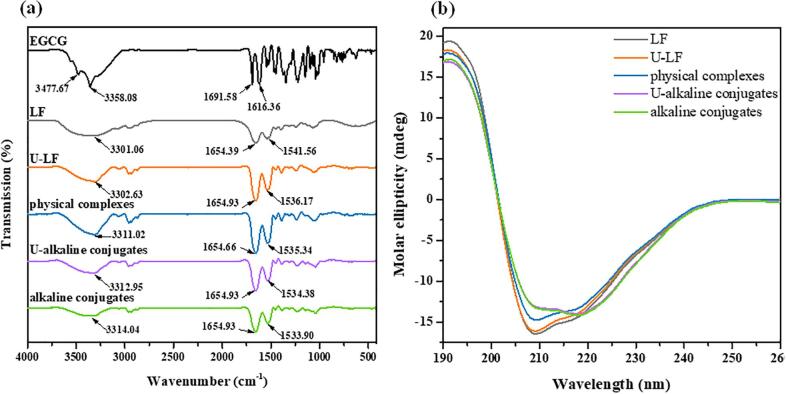
FTIR spectra (a) and CD spectra (b) of LF, U-LF, LF–EGCG physical complexes, U-alkaline conjugates and alkaline conjugates.

The FTIR spectra of different samples are shown in Fig. 2 (a). The typical characteristic peaks of EGCG at 1691 and 1616 cm^−1^ were attributed to the carbonyl stretching vibration, while the peaks at 3358 and 3477 cm^−1^ were attributed to the phenolic stretching vibration [38]. However, none of the typical characteristic peaks was found after the covalent or non-covalent grafting reaction between EGCG and LF, implying the interaction changed the molecular structure of EGCG.

The characteristic peaks of LF spectra were 3301.06 cm^−1^ (amide A), 1654.39 cm^−1^ (amide I), and 1541.56 cm^−1^ (amide II). The peak of LF at about 3301 cm^−1^ was associated with the stretching vibration of N—H coupled with hydrogen bonding. Compared to pure LF, both the covalent and non-covalent complexes showed a significant blue shift at 3301 cm^−1^. Meanwhile, the peaks of the spectra changed, which was consistent with hydrogen bond formation between EGCG and LF, and that the amino groups of LF were consumed. Our results are consistent with a previous study on covalent binding of pumpkin seed protein and gallic acid [39].

The peak of LF at about 1654.39 cm^−1^ was associated with the stretching vibration and the peak at 1541.56 cm^−1^ was associated with the N—H bending vibration and C—N stretching vibration. After alkaline treatment, the phenolic hydroxyl group in polyphenol was known to be oxidized to o-quinone, which could react with amino or sulfhydryl side chains of protein to form C—N or C—S covalent bonds [40]. For the amide I band, the peak value of the covalent/non-covalent complexes was slightly different from LF alone. However, in the amide II band, the peak value was prominently red-shifted from 1541.56 cm^−1^ (LF) to 1535.34 cm^−1^ (physical complexes), 1534.38 cm^−1^ (U-alkaline conjugates) and 1533.90 cm^−1^ (alkaline conjugates). These results imply that EGCG interacts with the C=O and C—N groups in LF to form non-covalent and covalent complexes.

#### 3.1.4 Far-UV circular dichroism (CD) spectroscopy

To evaluate the effect of ultrasonic treatment and EGCG covalent/non-covalent binding on the structure of LF, the secondary structure changes of LF were characterized using far-UV CD spectroscopy. As depicted in Fig. 2 (b), a broad negative peak ranging from 209 to 210 nm was observed in LF, which represented a typical α-helical structure. When EGCG was non-covalently bound to LF, the peak intensity of the sample decreased slightly but did not shift significantly. In contrast, when EGCG was covalently grafted to LF, there was a noticeable decrease in the peak intensity, a distinct red shift was observed, and a new peak was seen at 218 nm. In addition, the ultrasonic treatment also made the curves shift up slightly. The above results indicated that the ultrasonic treatment and polyphenol covalent/non-covalent modification would have varying degrees of effects on the secondary structure of LF.

The content of α-helix, β-sheet, β-turn, and random coil in the samples were evaluated using an online program (Dichroweb). As shown in Table 2, compared to pure LF, the ultrasonic treatment and EGCG addition both reduced the α-helix content of the protein. The U-alkaline conjugates exhibited the most significant change: the α-helix content decreased from 22.9 % to 19.4 %, while the random coil content increased from 26.6 % to 28.1 %. In addition, the extent of the changes in the secondary structure of the LF molecules depended on the processing method. Both ultrasonic treatment and EGCG non-covalent modification promoted a net change from α-helix to β-sheet. In contrast, the EGCG covalent modification promoted a net change from α-helix to random coil. The α-helical regions are mainly maintained by intrachain hydrogen bonds formed by carbonyl oxygen (C=O) and amino hydrogen (N—H) groups. The main secondary bonds that maintain the β-sheet are interchain hydrogen bonds formed by alternating peptide bonds between adjacent peptide chains [40], [41]. The change in secondary structure of the U-LF may be partly due to the cavitation, mechanical and thermal effects associated with sonication, which disrupted the hydrogen bonds, leading to a transformation of α-helix to β-sheet structures [42], [43]. In addition, the change in the physical complexes could be attributed to the fact that EGCG would interact with amino acid residues on the surface of LF to form complexes through hydrogen bonding, which would also lead to an α-helix to β-sheet transformation. These results are in accordance with previous reports [44], [45]. Compared to LF alone, the covalent binding of EGCG promoted a net change from α-helix to random coil. This result suggests that covalent binding of EGCG altered the stability of the LF molecular structure. In particular, hydrophobic amino acids in the protein (such as tryptophan and tyrosine) may have exposed to a more hydrophilic environment and participated in the reaction with polyphenols through C—N bonds, which could lead to an increase in the random coil content [46]. In this study, the largest change in the secondary structure of the proteins was observed for the combined treatments of ultrasound and polyphenol addition.

**Table 2:** The secondary structure fractions of LF, U-LF, LF–EGCG physical complexes, U-alkaline conjugates and alkaline conjugates determined by analysis of CD spectra.

**Sample Name**	**Content (%)**			
	**α-helix**	**β-sheet**	**β-turn**	**random coil**
LF	22.9	29.5	21.2	26.6
U-LF	21.4	31.8	21.3	25.5
physical complexes	20.6	32.7	21.1	25.7
U-alkaline conjugates	19.4	29.9	22.5	28.1
alkaline conjugates	20.5	29.4	22.8	27.5

### 3.2 DPPH free radical scavenging activity of LF–EGCG conjugates

**Fig. 3:**
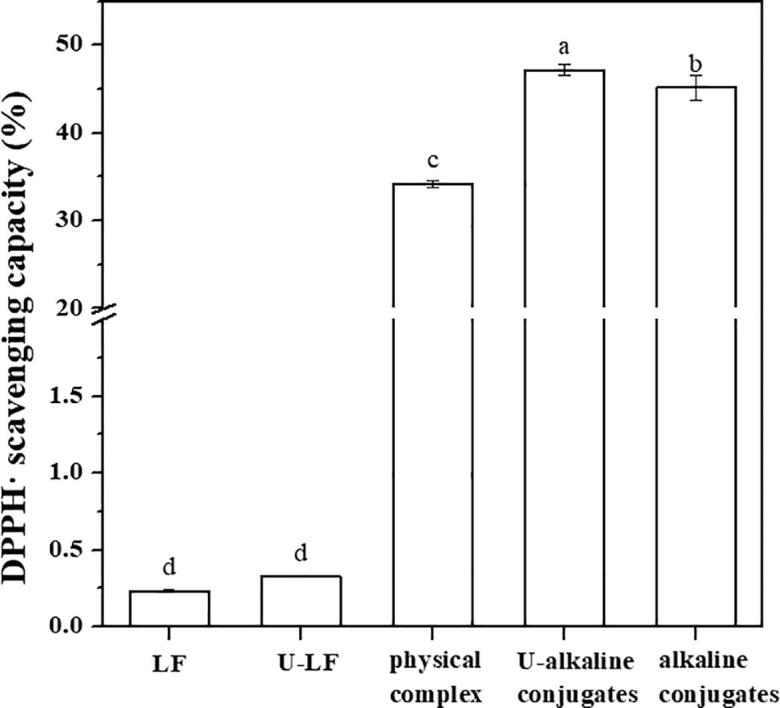
The DPPH free radical scavenging activity of LF, U-LF, LF–EGCG physical complexes, U-alkaline conjugates and alkaline conjugates. Different lowercase letters represent a significant difference (*p* < 0.05).

As shown in Fig. 3, the addition of EGCG significantly enhanced the DPPH free radical scavenging activity of the LF samples. At a concentration of 0.05 mg/mL, the scavenging activity of pure LF was only 0.23 %. The U-alkaline conjugates had the highest DPPH free radical scavenging capacity with a value of 47 %, followed by the alkaline conjugates with a value of 45 %. These results suggest that the antioxidant activity of LF could be improved by the covalent binding of EGCG, which could be attributed to the excellent free radical scavenging activity and metal ion chelating ability of this polyphenol [47]. Other researchers have also reported that the conjugation of proteins to tea polyphenols (*e.g.,* catechins, EGCG, and EGC) increased the DPPH free radical scavenging capacity of the proteins [9], [48].

The scavenging capacity of the DPPH free radicals was 34 % for the physical complexes, which suggested that some of the antioxidant EGCG did become attached to the LF. The disparity in DPPH free radical scavenging efficiencies of the different samples might be due to differences in the number and location of the polyphenols attached to the protein molecules for the different treatment methods. This hypothesis is consistent with the measured polyphenol binding equivalents shown in Table 1. The DPPH free radical scavenging rate of U-LF increased slightly compared to LF (Fig. 3), which may have occurred because the cavitation forces generated during sonication promoted partial protein unfolding. More aromatic amino acids (like tryptophan) may be exposed, which have antioxidant activity because of their participation in the direct transfer of electrons [20].

### 3.3 Stability of algae oil emulsions during storage

#### 3.3.1 Surface potential

**Fig. 4:**
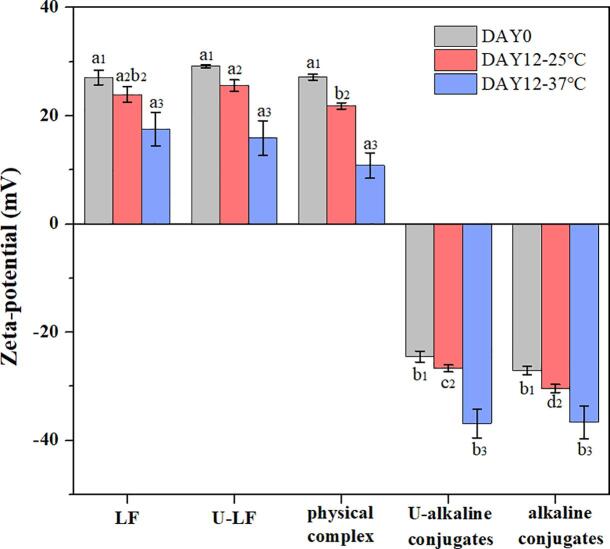
The zeta-potential of the emulsions stabilized by different samples after 0 and 12 days of storage at 25 and 37 °C. In the group of the same storage conditions (with the same column color), different lowercase letters with the same numeric corner scale (1, 2, and 3) represent a significant difference (*p*<0.05).

The zeta-potential is a measure of the effective surface potential of charged particles in solutions, which plays a strong role in determining the aggregation stability of emulsion droplets because it influences the magnitude of the electrostatic interactions acting between them [49]. For this reason, the zeta-potential changes of emulsion droplets stabilized by different emulsifiers were monitored after 0 and 12 days of storage at 25 and 37 °C under neutral conditions (Fig. 4). At 0 day, the zeta-potential of oil droplets coated by LF, U-LF, and LF–EGCG physical complexes were all positive being +27.1 ± 1.3, +29.2 ± 0.3, and + 27.2 ± 0.5 mV, respectively. This relatively high positive charge can be attributed to the fact that the pH was well below the isoelectric point of the LF molecules (pI: 8.0 ∼ 8.5) [50]. The ultrasonic treatment or the formation of non-covalent bonds with EGCG had little effect on the zeta-potential of the LF-coated oil droplets. However, the emulsions prepared by the LF–EGCG conjugates had negative zeta-potentials at 0 day. This effect can be attributed to the fact that the covalent binding of EGCG to LF reduced the isoelectric point of the protein to around 4.5 [18].

A comparison of the zeta-potential measurements of the emulsions after storage at 25 and 37 °C for 0 or 12 days showed that there was a decrease in positive charge (LF, U-LF, or physical complexes) or an increase in negative charge (conjugates) after storage. These changes are consistent with the generation of anionic species during storage that accumulate at the oil–water interface. A potential source of these anionic species is the auto-oxidation of the unsaturated fatty acids in the emulsions during storage, which generated hydroperoxides and their cleavage products. These results are consistent with those reported in a previous study in oil-in-water emulsions undergoing oxidation [51].

The increase in the absolute value of the zeta-potentials of the emulsions stabilized by the two kinds of LF–EGCG conjugates should lead to a stronger electrostatic repulsion between the emulsifier-coated droplets, thereby reducing their tendency to aggregate [52]. In contrast, the decrease in the absolute value of the zeta-potentials of the emulsions stabilized by pure proteins or physical complexes would be expected to result in a decrease in the electrostatic repulsion between the emulsifier-coated droplets, thereby increasing their propensity to aggregate [53]. A discussion of the aggregation behavior of these systems is included in the following section.

#### 3.3.2 Confocal laser scanning microscopy (CLSM)

**Fig. 5:**
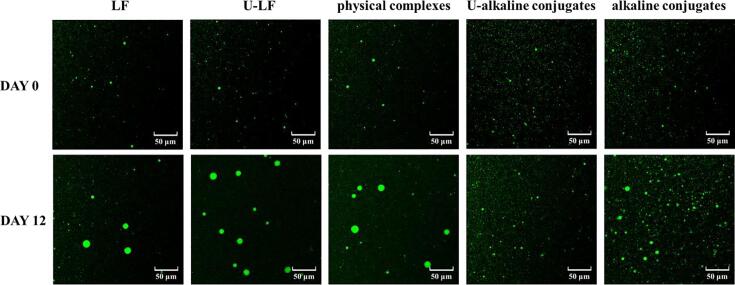
The CLSM of the emulsions stabilized by different samples after 0 and 12 days of storage at 37 °C.

CLSM was used to observe changes in the microstructure of the emulsions stabilized by the different emulsifiers for 12 days at 37 °C (Fig. 5). This higher storage temperature was used to accelerate the physical destabilization of the emulsions. The oil phase was stained with Nile Red, which appeared green at an excitation wavelength of 488 nm. The proteins were stained with Fast Green, which appeared red at an excitation wavelength of 633 nm.

Initially, all the emulsions contained relatively small oil droplets that were evenly dispersed throughout the samples. There was clearly an increase in droplet aggregation in all emulsions after 12 days storage (Fig. 5), but the magnitude of the effect depended on emulsifier type. In general, the emulsions appeared to have undergone coalescence during storage because there was an increase in the dimensions of the individual oil droplets. The degree of coalescence was higher in the emulsions stabilized by LF, U-LF, and the physical complexes than in those stabilized by the conjugates. This effect was probably due to the higher surface potential of the conjugates-coated oil droplets, which generated a stronger electrostatic repulsion that prevented the droplets coming close together during storage. Moreover, as mentioned earlier the absolute value of the surface potential of the emulsions stabilized by LF, U-LF, and physical complexes decreased during storage (Fig. 4), which would have progressively weakened the electrostatic repulsion between them. In contrast, the absolute value of the surface potential of the emulsions stabilized by the conjugates increased during storage (Fig. 4), thereby progressively increasing the electrostatic repulsion.

#### 3.3.3 Lipid oxidation stability of emulsions

Finally, the resistance of the emulsions to lipid oxidation was measured during storage by measuring a primary oxidation product (hydroperoxide value) and a secondary oxidation product (TBARS).

**Fig. 6:**
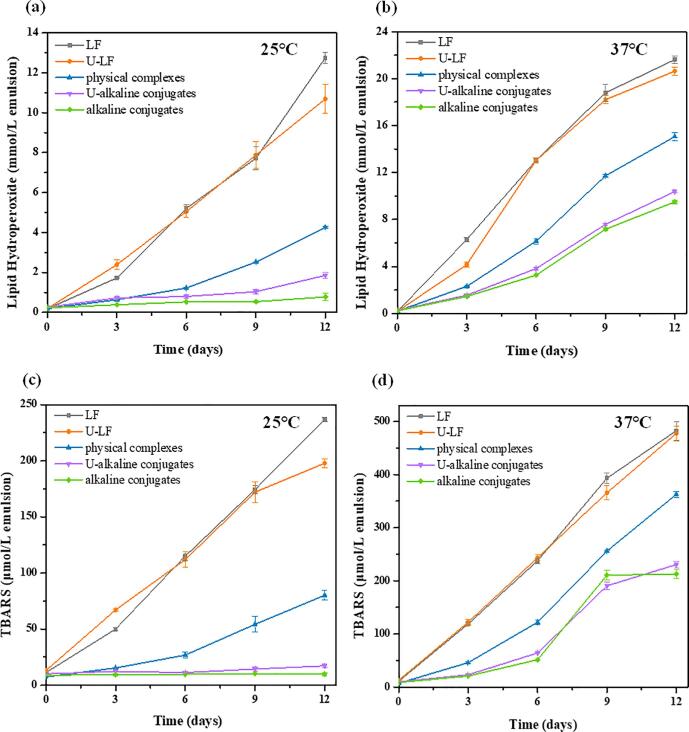
Evaluation of lipid hydroperoxide (a: 25°C, b: 37°C) and TBARS (c: 25°C, d: 37°C) in the emulsions stabilized by different samples during 12 days of storage.

The formation of hydroperoxides in the emulsions stabilized by different samples was measured at 25 and 37 °C for 12 days storage. As shown in Fig. 6 (a and b), the hydroperoxide values increased gradually with increasing storage time in all emulsions, which is indicative of lipid oxidation. The rate of the increase in hydroperoxides was higher at the higher storage temperature, which is to be expected because the lipid oxidation reaction increases in the presence of heat. The rate of hydroperoxide formation was fastest in the emulsions stabilized by LF, slowest in the ones stabilized by the LF–EGCG conjugates, and intermediate in the ones stabilized by the LF–EGCG complexes. These results suggest that the presence of the polyphenols at the oil–water interface increased the antioxidant activity of the adsorbed proteins. Presumably, there was a greater number of polyphenol molecules present when they were covalently attached to the proteins than when they were physically attached. Comparing the oxidation rate of the LF and U-LF samples indicates that sonication of the protein did not have a major impact on its antioxidant activity.

A similar result was observed for the secondary oxidation products (Fig. 6c and 6d), which form after decomposition of the lipid hydroperoxides. In general, the TBARS content of the emulsions increased with increasing storage time, but the rate of the increase differed appreciably between samples. As expected, the rate of TBARS formation was higher for the higher storage temperature. Moreover, the oxidation rate decreased in the following order: LF > physical complexes > covalent conjugates. Again, these results suggest that covalently attaching polyphenols to the surfaces of the protein increased its antioxidant activity. Other researchers have also reported that the resistance of emulsions to lipid oxidation can be improved by attaching phenolic compounds to them [54], [55]. In addition, it was noted that the content of primary and secondary oxidation products was slightly higher in the emulsions stabilized by U-alkaline conjugates than that of alkaline conjugates. This result was probably due to the larger specific surface area (smaller particle size) of the U-alkaline conjugate-stabilized emulsions during storage. As a result, more of the oil phase would be in direct contact with the external environment. However, there was no significant difference between the content of the primary and secondary oxidation products in the emulsions stabilized by the two types of LF-EGCG conjugates during storage. Also, a smaller particle size would improve the resistance of the emulsions to aggregation and creaming during storage.

## 4 Conclusions

In summary, LF–EGCG conjugates were successfully synthesized using an ultrasound-assisted alkaline method. Compared to the conventional alkaline method, the ultrasound-assisted one shortened the reaction time from 24 h to 40 min without effecting the EGCG grafting equivalents. The antioxidant properties of LF significantly increased after covalent conjugation to EGCG. In addition, oil droplets coated by LF–EGCG conjugates were found to be much more resistant to aggregation and lipid oxidation than those coated by LF. The improved aggregation stability was attributed to an increase in the electrostatic repulsion between the emulsifier-coated oil droplets, whereas the improved oxidative stability was attributed to a relatively high concentration of antioxidant polyphenols at the droplet surfaces. This study shows that protein–polyphenol conjugates can be used as dual-function ingredients that act as both emulsifiers and interfacial antioxidants in emulsions, thereby improving both their physical and chemical stability. These antioxidant emulsifiers may therefore be useful for application in functional foods containing polyunsaturated lipids.

## Declaration of Competing Interest

The authors declare that they have no known competing financial interests or personal relationships that could have appeared to influence the work reported in this paper.

## Data Availability

Data will be made available on request.
